# Stochastic modeling of injection induced seismicity based on the continuous time random walk model

**DOI:** 10.1038/s41598-024-55062-0

**Published:** 2024-02-28

**Authors:** Georgios Michas, Filippos Vallianatos

**Affiliations:** 1 Section of Geophysics – Geothermics, Department of Geology and Geoenvironment, National and Kapodistrian University of Athens, Athens, Greece; 2 Institute of Physics of Earth’s Interior and Geohazards, UNESCO Chair on Solid Earth Physics and Geohazards Risk Reduction, Hellenic Mediterranean University Research Center, Crete, Greece

**Keywords:** Continuous time random walk model, Time fractional diffusion equation, Injection induced seismicity, Cooper Basin, Anomalous diffusion, Geophysics, Seismology, Nonlinear phenomena, Statistical physics

## Abstract

The spatiotemporal evolution of earthquakes induced by fluid injections into the subsurface can be erratic owing to the complexity of the physical process. To effectively mitigate the associated hazard and to draft appropriate regulatory strategies, a detailed understanding of how induced seismicity may evolve is needed. In this work, we build on the well-established continuous-time random walk (CTRW) theory to develop a purely stochastic framework that can delineate the essential characteristics of this process. We use data from the 2003 and 2012 hydraulic stimulations in the Cooper Basin geothermal field that induced thousands of microearthquakes to test and demonstrate the applicability of the model. Induced seismicity in the Cooper Basin shows all the characteristics of subdiffusion, as indicated by the fractional order power-law growth of the mean square displacement with time and broad waiting-time distributions with algebraic tails. We further use an appropriate master equation and the time-fractional diffusion equation to map the spatiotemporal evolution of seismicity. The results show good agreement between the model and the data regarding the peak earthquake concentration close to the two injection wells and the stretched exponential relaxation of seismicity with distance, suggesting that the CTRW model can be efficiently incorporated into induced seismicity forecasting.

## Introduction

Injection-induced seismicity has recently drawn much scientific, societal, and political attention owing to the recent surge of globally reported events^1–3^. Fluid injections into the subsurface, associated with wastewater disposal, hydraulic fracturing, carbon dioxide (CO_2_) sequestration, or geothermal heat extraction operations at energy production sites, can reduce the shear strength of prestressed faults, triggering earthquakes^4,5^. Although fluid injections may routinely cause none or some minor microseismicity in the injection area^6^, in other cases, intense microseismicity or even larger magnitude events with damaging effects can be induced^2,7^. While such incidents have long been known^4,8,9^, the increasing demand for low-carbon energy production, the rapid advancement of new drilling and exploitation technologies and new larger-scale industrial projects and injection sites will likely cause induced seismicity cases to proliferate.

The main driving mechanism of injection-induced seismicity is attributed to an increase in pore pressure in proximity to the injection area, which can reduce the effective normal stress acting across fault planes, allowing them to slip^4^. However, poroelastic stresses acting on the broader injection area^10^, stress interactions between induced events^11^, thermoelastic-induced stress changes^12^ and pore-fluid pressure-driven aseismic slip^13,14^ can also play a prime role in inducing earthquakes. In addition, the spatiotemporal occurrence of induced events can be erratic, expanding in larger than expected distances away from the injection site at timescales that may vary from days up to months or even years^15–17^. To effectively mitigate the associated risk, the main challenge is to better understand the in situ and operational conditions that may lead to larger and potentially hazardous events and to effectively delineate the spatiotemporal evolution of injection-induced seismicity.

Where, when and how many induced earthquakes may occur above a given threshold magnitude are generally obscure owing to the complexity of the injection site, the local geological conditions, the regional stress field, and the injection parameters^18^. Given the large heterogeneities within the Earth’s crust, the complex and frequently unknown fracture and fault network and the highly anisotropic and dynamically changing permeability field that may regionally vary over several orders of magnitude, the front of the pore-fluid pressure increase can migrate at large distances away from the injection site, bringing critically stressed faults closer to failure, even in the far field^15–18^. In addition, poroelastic stresses can be even more remote, expanding well outside the hydraulically connected area where the pore-fluid pressure increases^16,19^. Stress interactions and thermal effects further add to the complexity of the physical processes occurring in the subsurface following fluid injection, making any prognostic evaluation of the evolution of induced seismicity extremely difficult.

Considering the complexity that may characterize the evolution of injection-induced seismicity, the large uncertainties regarding the in situ physical properties in the subsurface and the wide range of spatial and temporal scales that may appear, we consider a purely stochastic framework that generates the essential properties of the process. Along this line, we build on the well-established continuous-time random walk (CTRW) theory and the fractional diffusion equation to map the spatiotemporal evolution of injection-induced seismicity and to make predictions regarding the conditional probabilities of earthquake occurrence. To test and demonstrate the applicability of the model, we use data from the 2003 and 2012 hydraulic stimulations that were performed at the Cooper Basin geothermal field (South Australia). Fluid injections in the Cooper Basin, aimed to enhance the hydraulic permeability of the hot granitic basement and develop an enhanced geothermal system (EGS), induced thousands of microearthquakes and provided some of the most prolific seismic datasets in EGSs. The analysis and results within the CTRW framework demonstrate the applicability of the model in such cases of anomalous earthquake diffusion, as discussed analytically in the following section.

## Methods

Within the CTRW context, we consider seismicity as a point process in time and space marked by the magnitude of the event. We also consider that, starting from the origin (*x*_0_ = 0, *t*_0_ = 0), seismicity undergoes a random walk in time and space, where each new event site is the new position of the random walk that occurs after waiting some time *τ* at the previous site. Then, we calculate the 3D Euclidean distance *x*(*t*) between each event and the origin, taken as the injection point, and estimate the mean square displacement (MSD) $\left\langle {x^{2} \left( t \right)} \right\rangle$ of seismicity with time according to^20^:

1 $$\left\langle {x^{2} \left( t \right)} \right\rangle = \frac{1}{N}\mathop \sum \limits_{n = 1}^{N} x_{n}^{2} \left( t \right).$$

where *N* is the total number of events and *x*_*n*_(*t*) is the distance of the *n*th event from the origin that occurred at time *t* from the initiation of injection. In various complex systems, the MSD grows nonlinearly with time *t*, frequently taking the form of the power-law function^21,22^:

2 $$\left\langle {x^{2} \left( t \right)} \right\rangle \propto t^{a} .$$

In such cases, anomalous diffusion arises for *a* ≠ 1, distinguishing superdiffusion for *a* > 1 and subdiffusion for 0 < *a* < 1, while normal diffusion is recovered for *a* = 1. In the following, we use the term “anomalous” to characterize the diffusive process that deviates from the linear growth of the MSD with time (*a* ≠ 1) and eventually from the linear diffusion equation (see “Supplementary Methods”).

In addition, the subdiffusive regime in the CTRW model, which is the relevant one for the cases that we study (see “Results” section), corresponds to broad waiting-time distributions with divergent characteristic waiting times *T* (see “Supplementary Methods”). To approximate the scaling behavior of the waiting-time distribution, we use the *q*-generalized gamma function that has been found suitable for nonstationary earthquake time series^23^. The *q*-generalized gamma function reads as follows:

3 $$f\left( \tau \right) = C\left( {\frac{\tau }{{\tau_{0} }}} \right)^{\gamma - 1} \exp_{q} \left( { - \frac{\tau }{{\tau_{0} }}} \right),$$

where *C* is a normalization constant, *τ*_0_ is a positive scaling parameter and *γ* is the scaling exponent. The last term on the right-hand side of Eq. (3) is the *q*-exponential function defined as follows:

4 $$\exp_{q} \left( x \right) = \left[ {1 + \left( {1 - q} \right)x} \right]^{{1/\left( {1 - q} \right)}}$$

The *q*-exponential function is associated with generalized statistical mechanics, as it maximizes the nonadditive entropy *S*_*q*_ under appropriate constraints^24^, while its applicability to earthquake dynamics has been demonstrated in various studies (e.g.,^25,26^ and references therein). For *q* > 1, the *q*-exponential function exhibits asymptotic power-law behavior, while at the *q* → 1 limit, the ordinary exponential function is exactly recovered. Hence, for *q* > 1, the *q*-generalized gamma function (Eq. (3)) exhibits double power-law behavior, where short and large *τ* scale as power laws according to $\sim \tau^{\gamma - 1}$ and $\sim \tau^{{\left( {1 - \gamma } \right)/\left( {1 - q} \right)}}$, respectively, while for *q* → 1, the ordinary gamma function is exactly recovered^23^.

Moreover, the subdiffusive regime of the CTRW model is interchangeable with the time-fractional diffusion equation (TFDE)^27^:

5 $$\frac{\partial }{\partial t}P\left( {x,t} \right) = {}_{0}D_{t}^{1 - a} K_{a} \frac{{\partial^{2} }}{{\partial x^{2} }}P\left( {x,t} \right),$$

where *P*(*x*,*t*) is the probability density function of the walker being at some position *x* after time *t*, *K*_*a*_ the generalized diffusion coefficient and ${}_{0}D_{t}^{1 - a}$ the Riemann–Liouville fractional operator of order *1–a* (0 < *a* < 1). In the “Supplementary Methods”, we describe the derivation of the TFDE and its asymptotic solution in terms of the standard theorem of the Fox hypergeometric functions. For large *x* $\left( {x > \sqrt {K_{a} t^{a} } } \right)$, the asymptotic behavior of *F*(*x*,*t*), i.e., the probability density of the number of earthquakes that have just occurred at some position *x* from the origin after some time *t*, is given by^20,28^:

6 $$F\left( {x,t} \right)\sim \frac{{\left( {\tau^{\prime}} \right)^{ - a} }}{{\sqrt {{K}_{\alpha } } t^{{1 - \left( {a/2} \right)}} }}\left( {\frac{\left| x \right|}{{\sqrt {K_{a} t^{a} } }}} \right)^{{d\left( {1 - a} \right)/\left( {2 - a} \right)}} \times \exp \left[ { - \left( {1 - \frac{a}{2}} \right)\left( \frac{a}{2} \right)^{{a/\left( {2 - a} \right)}} \left( {\frac{\left| x \right|}{{\sqrt {K_{a} t^{a} } }}} \right)^{{2/\left( {2 - a} \right)}} } \right]$$

where *a* is the diffusion exponent (Eq. (2)), *d* is the spatial dimension and $\tau^{\prime} = T\left( {{\Gamma }\left( {1 - a} \right)} \right)^{1/a}$ (for *a* < 1). The previous asymptotic solution of the TFDE model indicates a power-law increase in *F*(*x*,*t*) for short *x*, with a slope of $d\left( {1 - a} \right)/\left( {2 - a} \right)$, while the second term on the right-hand side of Eq. (6) indicates the asymptotic exponential decay of *F*(*x*,*t*) for long *x*.

## Results

### Hydraulic stimulations and induced seismicity in the Habanero field

Hydraulic stimulations at the Cooper Basin initiated in early November 2003 with the main stimulation of the Habanero-1 well. Fluid injections commenced on 30 November 2003 and lasted approximately 9.5 days (Supplementary Fig. S1). During stimulation, more than 20,000 m^3^ of water was injected into the 4421-m-deep well, indicating a distinct flow zone at a depth of ~ 4254 m^29^. Flow rates increased stepwise during the main stimulation period, reaching a maximum rate of ~ 45 L/s and wellhead pressure peak values of ~ 65 MPa (Supplementary Fig. S1). Fluid injections induced more than 28,000 microseismic events, with 15,709 recorded during the main stimulation period. Induced microseismicity showed a general tendency to migrate away from the well with continuation of injection (Supplementary Fig. S2), while the alignment of the events along a zone of vertical thickness ~ 100–150 m indicated a single fracture zone as the focus of microseismicity^29^.

The 4204-m-deep Habanero-4 well was hydraulically stimulated in 2012 to further extend the previous stimulated reservoir. Fluid injections commenced on 14 November 2012, while the main stimulation period lasted approximately 14 days, between 16 and 30th November 2012 (Supplementary Fig. S1). In total, 34,000 m^3^ of water was injected into the well, inducing more than 29,000 microseismic events during a 2-month period^30^. During the main stimulation of the well, the flow rate reached a maximum value of ~ 60 L/s, and the wellhead pressure reached a peak value of ~ 50 MPa (Supplementary Fig. S1). The recorded microseismicity reached 17,266 events, indicating a general tendency to migrate away from the injection point, particularly toward the north (Supplementary Fig. S2). Vertical location errors on the order of ~ 100 m, as well as image logs from the Habanero-3 well, indicate that a few-meter-thick single planar fault zone, shallowly dipping to the west—southwest, dominates the occurrence of fluid flow and induced seismicity in the Habanero field^30^.

To proceed with the analysis, we used the microseismic data collected during the main stimulation periods, i.e., 30/11–09/12/2003 for Habanero-1 and 16/11–30/11/2012 for Habanero-4 (Supplementary Fig. S1). We estimate the magnitude of completeness (*M*_c_) for each earthquake catalog using the nonparametric method of median-based analysis of the segment slope (mbass;^31^), which is considered more adequate for gradually curved frequency–magnitude distributions^32^. The implementation of the mbass method indicates an apparent breakpoint in the discrete frequency-magnitude distributions of the Habanero-1 and Habanero-4 earthquake catalogs at *M* –0.7 and *M* –0.6, respectively (Supplementary Fig. S3), which are further considered as the *M*_c_ for the two catalogs. For *M* ≥ *M*_c_, the final catalogs that are further used in the analysis consist of 9491 and 7708 events for Habanero-1 and Habanero-4, respectively.

### Earthquake diffusion properties

To quantify the rate of earthquake diffusion during the two hydraulic stimulations in terms of the MSD of induced seismicity with time (Eq. (2)), we take as the origin the intersection point of the open hole sections in the two wells and the distinct flow zones (− 4254 m, Habanero-1; − 4160 m, Habanero-4). We then calculate $\left\langle {x^{2} \left( t \right)} \right\rangle$ according to Eq. (1) for the events with *M* ≥ *M*_c_.

**Figure 1:**
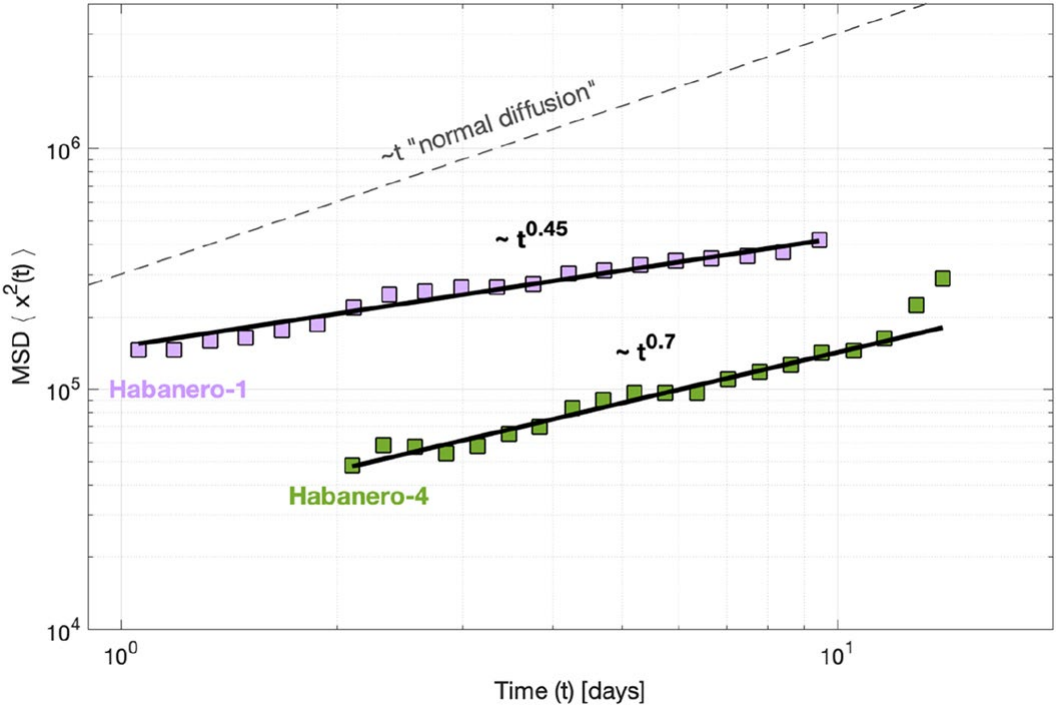
MSD of induced seismicity with time for Habanero-1 and Habanero-4 (filled squares), in logarithmically spaced bins and on double logarithmic axes. The solid lines represent the best-fitting solutions according to the power-law relationship of Eq. (2). The dashed line represents the trend (*a* = 1) for normal diffusion.

The MSD of induced seismicity with time for Habanero-1 and Habanero-4 is shown in Fig. 1 (for logarithmically binned data and on log–log axes). For both cases, the MSD grows almost continuously with time, highlighting the diffusive process. The increase in the MSD with time approximates the power-law relationship of Eq. (2), with diffusion exponents *a* = 0.45 ± 0.04 (R^2^ = 0.97) for Habanero-1 and *a* = 0.70 ± 0.05 (R^2^ = 0.98) for Habanero-4 (Fig. 1), with uncertainties referring to 95% confidence intervals. A lower than unity exponent *a*, observed for both Habanero-1 and Habanero-4, indicates anomalous diffusion of induced seismicity according to a subdiffusive process. The rate of earthquake diffusion, as quantified by the exponent *a*, is greater for Habanero-4 (*a* = 0.70) than for Habanero-1 (*a* = 0.45). Note, however, that the latter does not necessarily signify longer distances of induced events from the origin. For instance, in Fig. 1, we observe that the instantaneous spatial response of induced seismicity during the first days of injection is greater in Habanero-1 than in Habanero-4. For Habanero-4, we also observe that during the last two days of injection, the MSD departs from the trend ~ *t*^0.7^ and instead shows faster growth, as the northern part of the stimulated volume, located further away from the well, becomes seismically active (Supplementary Fig. S2).

### The waiting-time distribution

To validate the CTRW model and to extract further information regarding the temporal structure of induced seismicity during the two hydraulic stimulations, we investigate the distribution of waiting times *τ*, i.e., the time intervals between successive earthquakes, defined as *τ*_*i*_ = *t*_*i*+1_ – *t*_*i*_, with *t*_*i*_ being the time of occurrence of the *i*th event. To ensure homogeneity in the analysis, we only consider earthquakes with magnitudes *M* ≥ *M*_c_. We estimate the probability density *P*(*τ*) of waiting times *τ* by counting the number of *τ* that fall into logarithmically spaced bins and then normalize this value by dividing this number by the bin width and by the total number of counts so that the probabilities of occupation sum to one. The analysis is restricted to waiting times *τ* ≥ 10 s due to significant scatter that appears in the estimated *P*(*τ*) for *τ* < 10 s, particularly for the Habanero-4 earthquake catalog, which may bias the scaling analysis.

**Figure 2:**
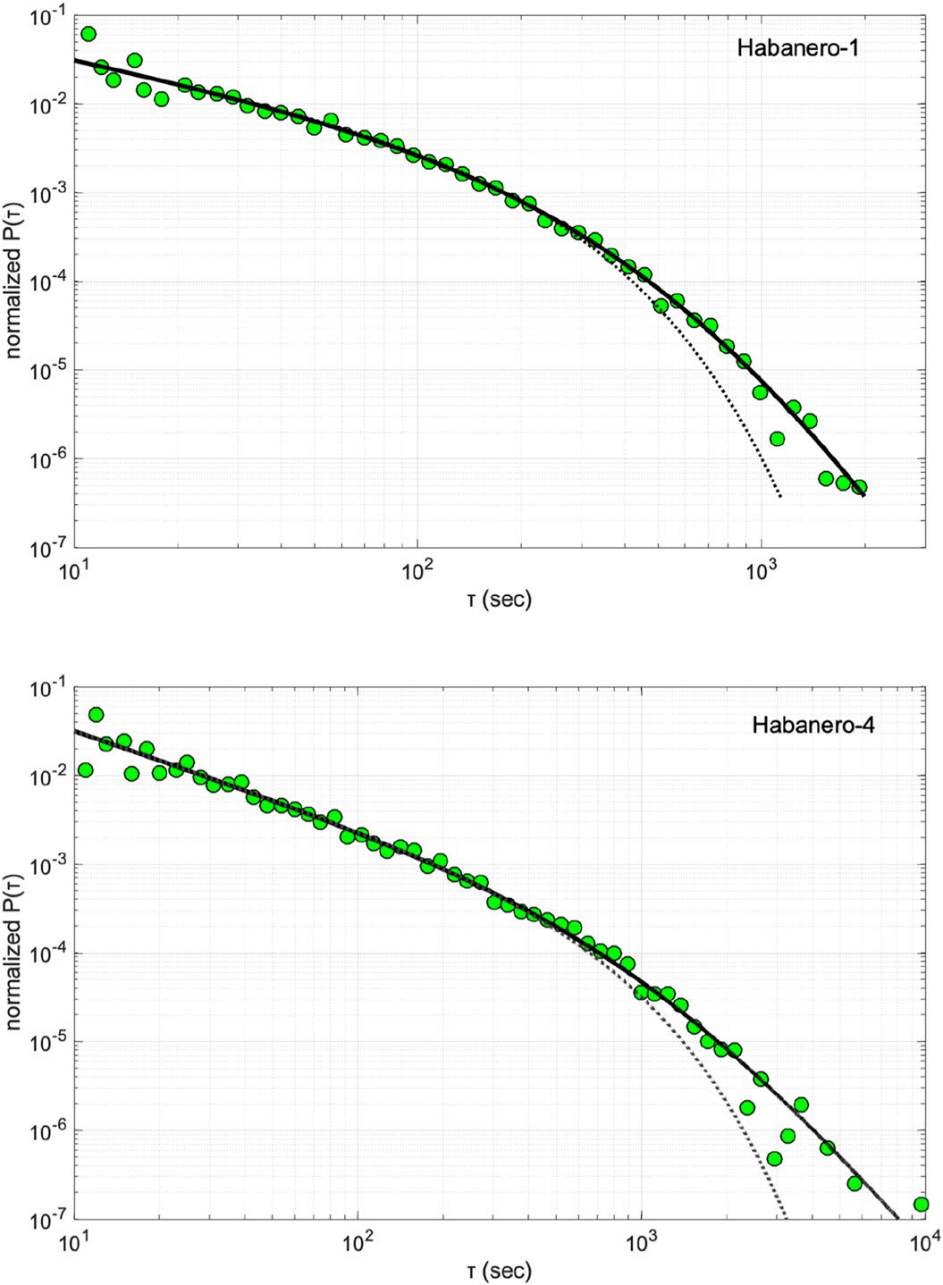
Normalized probability densities *P*(*τ*) of the waiting times *τ* (filled circles) on double logarithmic axes for Habanero-1 (top) and Habanero-4 (bottom). The model (solid line) represents Eq. (3) for the parameter values referred to in the main text, while the dotted lines the standard gamma function fitted to the data.

The normalized probability densities *P*(*τ*) for Habanero-1 and Habanero-4 are shown in Fig. 2. For both datasets, we observe a bimodal behavior of *P*(*τ*). For short *τ*, *P*(*τ*) decays slowly up to a characteristic waiting time, where a gradual crossover to faster decaying probabilities appears for larger *τ* (Fig. 2). We approximate this scaling trend with Eq. (3), using a weighted nonlinear least squares algorithm to estimate the model parameters and their associated uncertainties (95% confidence intervals). Regression yields the parameter values *C* = 0.21 ± 0.02, *γ* = 0.20 ± 0.03, *τ*_0_ = 131.21 ± 38.69 and *q* = 1.19 ± 0.07, with a root-mean-square error (RMSE) of 0.0043 for Habanero-1 and the values *C* = 0.38 ± 0.03, *γ* = –0.07 ± 0.03, *τ*_0_ = 459.63 ± 93.4 and *q* = 1.36 ± 0.08, with a RMSE of 0.0037, for Habanero-4 (Fig. 2). The observed *P*(*τ*) for both Habanero-1 and Habanero-4 can well be approximated with the *q*-generalized gamma function, contrasting with random Poissonian behavior and suggesting clustering effects at all time scales in the evolution of injection-induced seismicity in the Habanero field. We recall that Gaussian propagation is associated with asymptotic exponential scaling in the time domain^33^ so that a long-tailed waiting-time distribution with asymptotic power-law scaling is an additional manifestation of anomalous diffusion, particularly subdiffusion.

### Propagation of induced seismicity

In the previous sections, we established the subdiffusive character of the seismicity induced during the main stimulations of the Habanero-1 and Habanero-4 wells. In the current section, we proceed one step further to model the propagation of induced seismicity in space and time in terms of the pdf *F*(*x*,*t*), or else the concentration profile, which expresses the probability density of the number of earthquakes that have just occurred at some position *x* from the origin after some time *t* (see also “Supplementary Methods”). To model *F*(*x*,*t*), we use the time-fractional diffusion equation (TFDE) (Eq. (5)) and its asymptotic solution (Eq. (6)).

**Figure 3:**
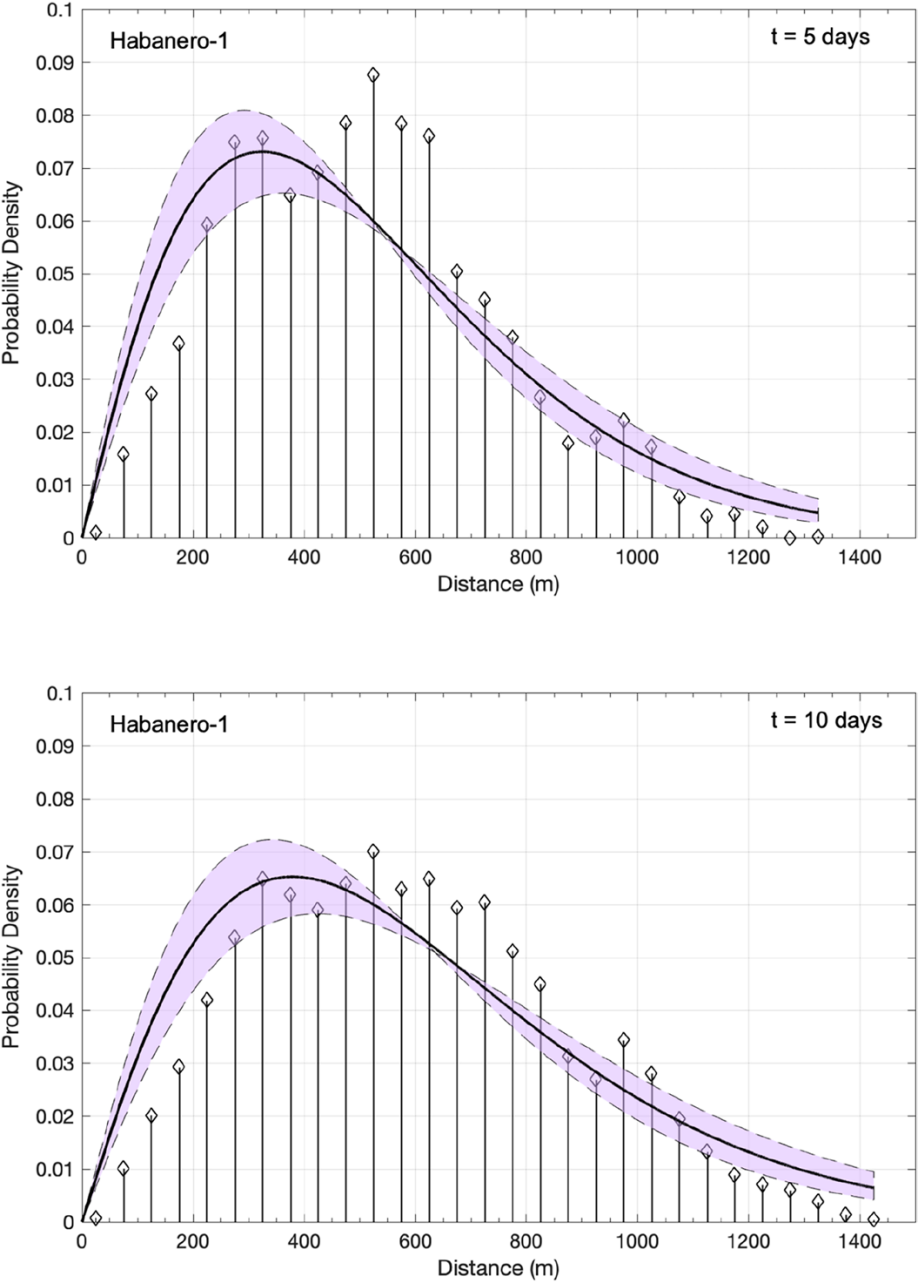
Normalized probability density of the absolute 3D distances between the induced events and the origin during the main stimulation of the Habanero-1 well, represented as a stem plot for *t* = 5 days (top) and *t* = 10 days (bottom). The solid line and the shaded area indicate the asymptotic solution of the TFDE (Eq. (6)) and the corresponding confidence intervals (see text), respectively.

**Figure 4:**
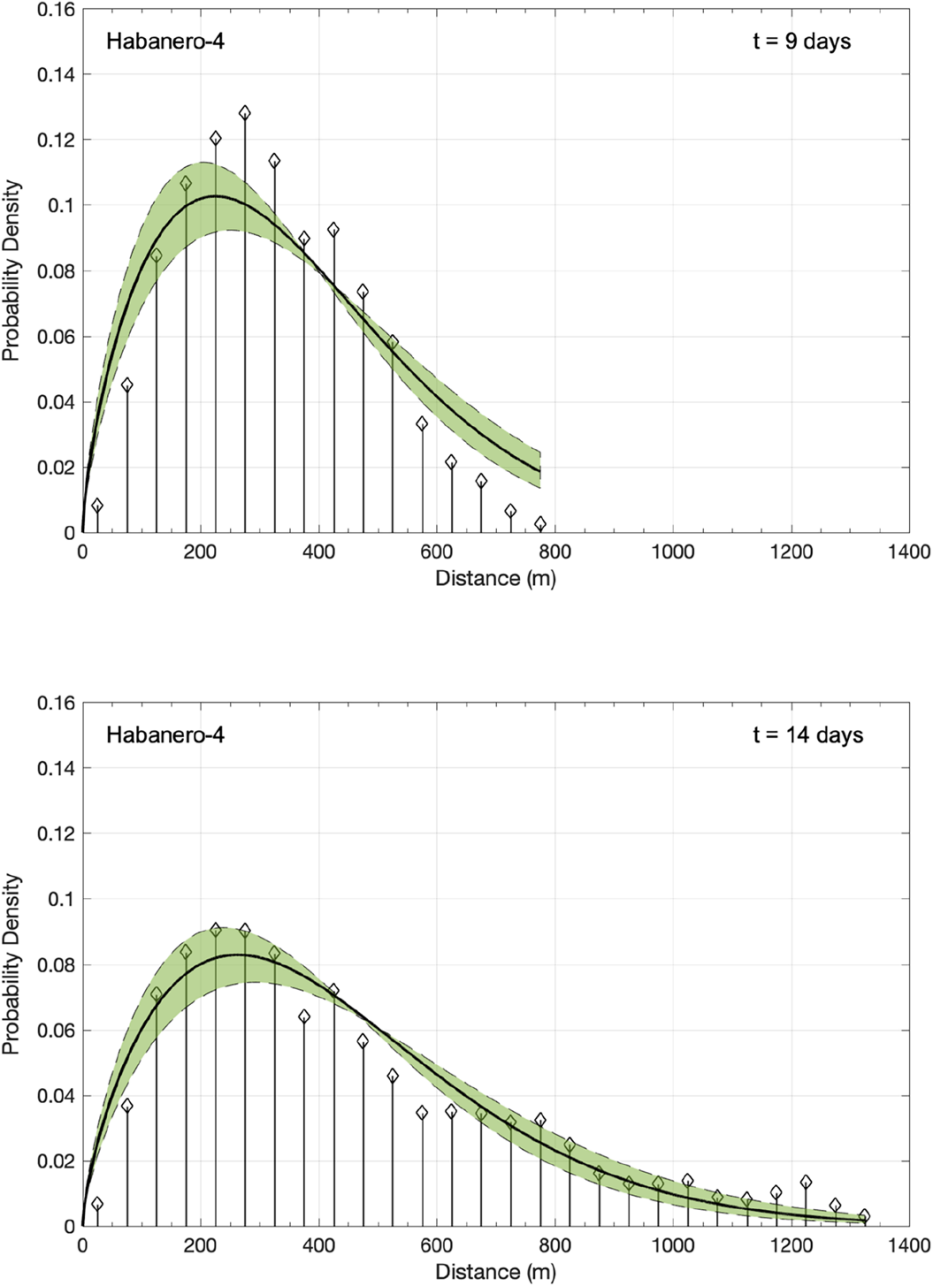
Normalized probability density of the absolute 3D distances between the induced events and the origin during the main stimulation of the Habanero-4 well, represented as a stem plot for *t* = 9 days (top) and *t* = 14 days (bottom). The solid line and the shaded area indicate the asymptotic solution of the TFDE (Eq. (6)) and the corresponding confidence intervals, respectively.

To estimate *F*(*x*,*t*), we construct the histogram of the absolute 3D distances between each induced event (for *M* ≥ *M*_c_) and the origin for various time periods. We then normalize *F*(*x*,*t*) such that $\smallint dxF\left( {x,t} \right) = 1$. The normalized probability densities *F*(*x*,*t*) for 50 m wide consecutive bins and for two time periods are shown in Fig. 3 for Habanero-1 and in Fig. 4 for Habanero-4. For Habanero-1, we estimate *F*(*x*,*t*) for *t* = 5 days and for *t* = 10 days, which mark approximately the time of occurrence of 50% of the induced events and the termination of injection, respectively, while for Habanero-4, we estimate *F*(*x*,*t*) for *t* = 9 days (45% of the induced events) and for *t* = 14 days (termination of injection). From Figs. 3 and 4, we can immediately verify for both cases the departure of *F*(*x*,*t*) from the classic bell-shaped Gaussian function that signifies normal diffusion (see also “Supplementary Methods”). Instead, we observe a peak closer to the origin, i.e., the injection point, and a tail that stretches toward greater distances.

To model the observed *F*(*x*,*t*) with the solution of the TFDE (Eq. (6)), we use the model parameters that emerged from the previous analysis of the waiting-time distribution and the MSD of seismicity with time. In particular, for Habanero-1, the mean waiting time was *T* = 89.42 s, and the diffusion exponent *a* = 0.45; for Habanero-4, the mean waiting time was *T* = 143.63 s and* a* = 0.70. In addition, we set the spatial dimensions to *d* = 3, as we analyze 3D distances between earthquake hypocenters and the origin. We then use the generalized diffusion coefficient *K*_*a*_ as the only free parameter to optimize the model. The nonlinear least squares algorithm was applied to optimize Eq. (6) for *K*_*a*_ and to estimate the associated 95% confidence intervals, yielding *K*_*a*_ = 23.67·10^3^ ± 6.1·10^3^ (for *t* = 10 days) for Habanero-1 and *K*_*a*_ = 8.42·10^3^ ± 2·10^3^ (for *t* = 14 days) for Habanero-4. As shown in Figs. 3 and 4, the model successfully captures the main features of earthquake occurrence, namely, the peak concentration close to the injection site, the narrowing and broadening of the peak with time and the stretched relaxation of seismicity with distance. However, some discrepancies are still present, as we can observe for Habanero-1 (Fig. 3), where the model fails to predict the second greater peak at ~ 500–550 m for *t* = 5 days and the quite broad peak (~ 300–650 m) for *t* = 10 days. For Habanero-4 and for *t* = 9 days, the model slightly underpredicts the peak earthquake concentration and overpredicts the concentration of distant events, while for *t* = 14 days, it successfully predicts the peak concentration and the spatial relaxation of seismicity (Fig. 4).

### Propagation along the principal axes

To account for anisotropic hydraulic diffusivities inside the stimulated volumes, as implied by the elongation of the seismicity clouds along a general NS direction (Supplementary Fig. S2), and to further validate the applicability of the model, we downscaled the analysis to 1D and studied the spatial propagation of seismicity along the principal axes of an ellipsoid that best fit the seismicity clouds. The latter enables the estimation of the spatial density and the average propagation of seismicity in terms of the centered MSD along the main spreading directions. To perform this analysis, we initially use the statistical method of principal component analysis (PCA) to estimate the principal components of the seismicity cloud and to define the principal axes of the ellipsoid.

The approximate 2D geometry of the seismicity cloud during stimulation of the Habanero-1 and Habanero-4 wells^29,30^ is further confirmed with PCA, as the eigenvalues of the two main principal components can explain more than 98.5% of the total observed spatial variance of seismicity in 3D space. Hence, we can approximate the spatial extent of seismicity clouds with an ellipse rather than an ellipsoid, assuming that 95% of all events are located inside this ellipse. The estimated ellipses for the two seismicity clouds, centered to the two wells, define areas of approximately 3.27 km^2^ and 3 km^2^, with the first principal axes oriented 22.5° and 354° from the North for Habanero-1 and Habanero-4, respectively (Supplementary Fig. S4).

We rotate the seismicity clouds around the two origin points so that the x- and y-axes coincide with the principal axes. We then calculated the 1D absolute distances of the rotated events from the two origin points to estimate the MSD and the spatial density of the observed seismicity along the two principal axes. As previously described, the MSD grows as a power-law with time with diffusion exponents less than unity in all cases. In particular, for Habanero-1, *a* = 0.39 ± 0.06 and *a* = 0.43 ± 0.06 along the first and second principal axes, respectively, for *t* in the range between 2 and 10 days (Supplementary Fig. S5). For Habanero-4, *a* = 0.68 ± 0.09 and *a* = 0.83 ± 0.11 along the first and second principal axes, respectively, for *t* > 3 days (Supplementary Fig. S5). By further setting *d* = 1 in Eq. (6) and by using *K*_*a*_ as the only free parameter to optimize the model, we estimate the asymptotic solution of the TFDE fitted to the data. As previously described in 3D space, the model manages to capture the main properties of earthquake progression in time and space, particularly for Habanero-4 (Supplementary Figs. S6 and S7).

**Figure 5:**
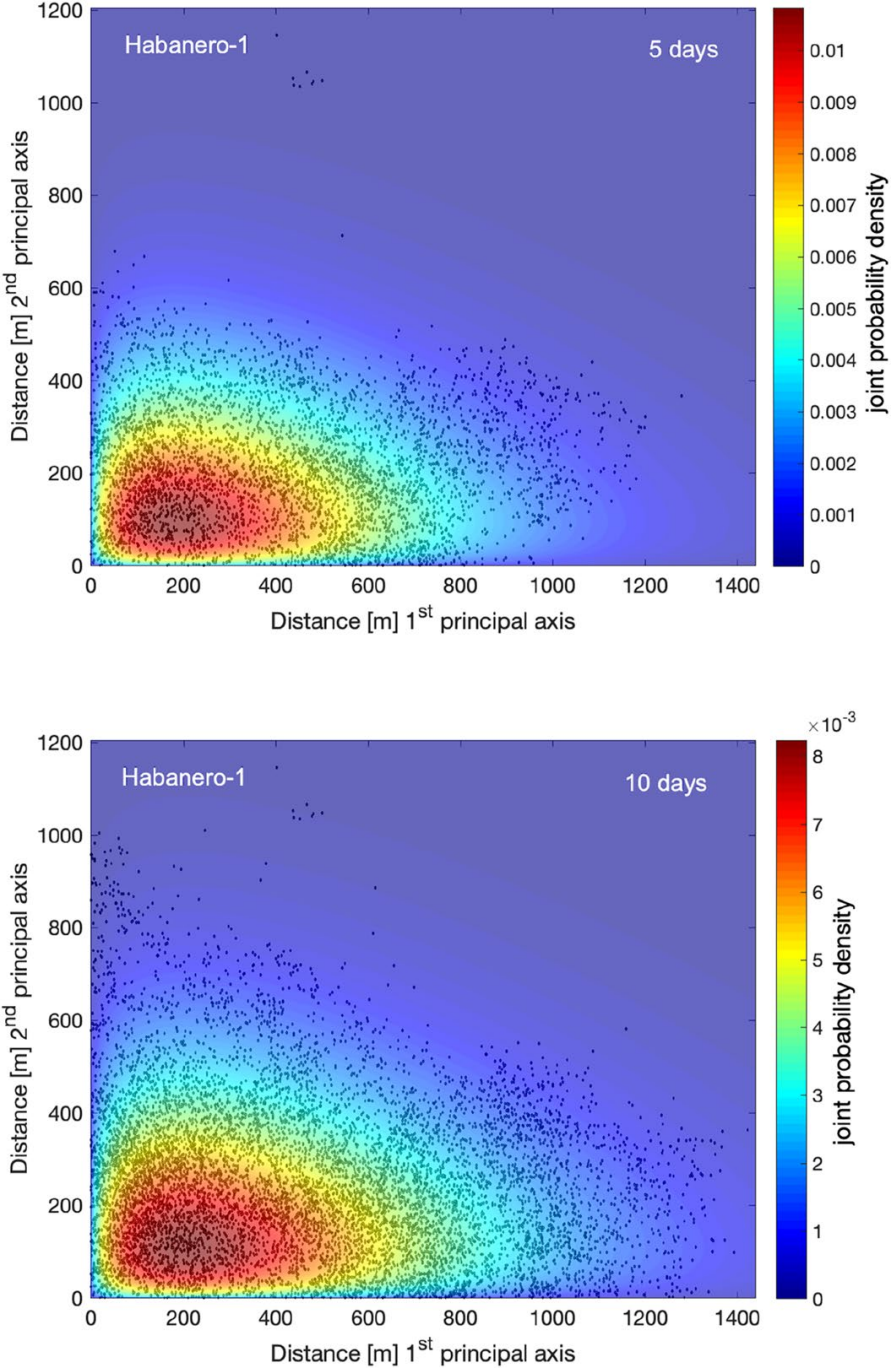
Joint probability density of earthquake occurrence at Habanero-1 during the main stimulation period, after 5 (top) and 10 days (bottom) from initiation of injections. The absolute earthquake locations along the two principal axes, estimated with PCA, are shown with black dots. Figures were created with MATLAB.

**Figure 6:**
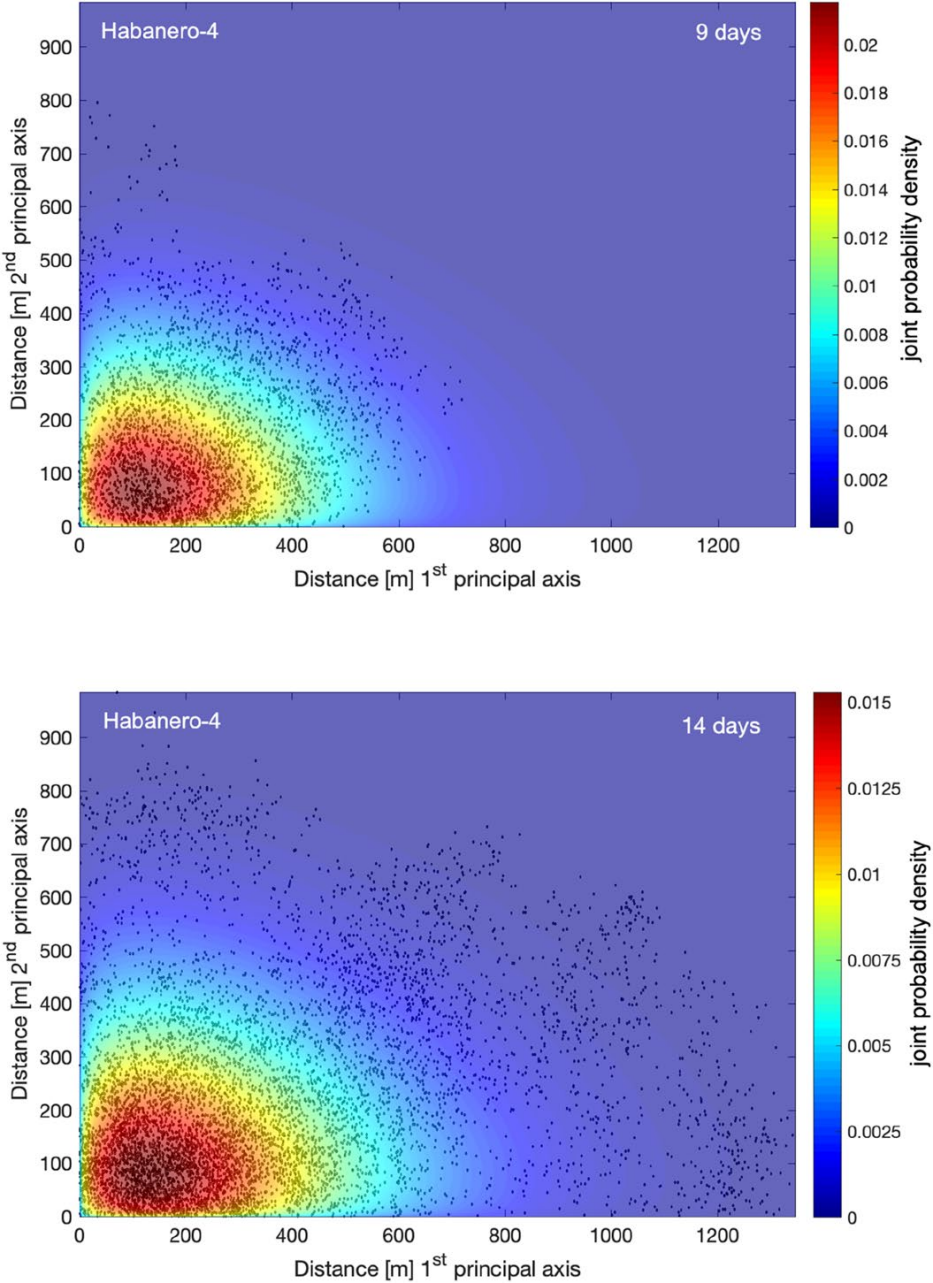
Joint probability density of earthquake occurrence at Habanero-4 during the main stimulation period, after 9 (top) and 14 days (bottom) from initiation of injections. The absolute earthquake locations along the two principal axes, estimated with PCA, are shown with black dots. Figures were created with MATLAB.

Furthermore, we use the estimated pdfs *F*(*x*,*t*) to model the propagation of induced seismicity in 2D space with Eq. (6). In this case, we estimate the joint probability density function as the product of the marginal pdfs *F*(*x*,*t*). The results are presented in Figs. 5 and 6 for Habanero-1 and Habanero-4, respectively, and for two time periods. In particular, Figs. 5 and 6 show the spatial distributions of induced seismicity along the two principal axes of the ellipse that resulted from PCA and the joint probability density of earthquake occurrence according to Eq. (6). For Habanero-1 and for both *t* = 5 days and *t* = 10 days, the model successfully predicts the area of greater earthquake occurrence close to the origin, the greater extent of seismicity along the first principal axis and the decay of earthquake occurrence with distance from the origin (Fig. 5). A similar image was obtained for Habanero-4 (Fig. 6). In this case, for both *t* = 9 days and *t* = 14 days, the model successfully predicts the peak earthquake occurrence close to the origin, which remains nearly fixed with increasing time, and the gradual decay of seismicity with distance from the origin, while it seems to slightly underpredict the occurrence of seismicity at distances greater than ~ 900 m along the first principal axis for *t* = 14 days (see also Supplementary Fig. S7).

## Discussion

Injection-induced seismicity represents a major challenge for the development of EGSs, and its effective management is vital for public consensus and successful completion^34,35^. Under these conditions, deciphering the actual physical mechanisms that underlie the spatiotemporal evolution of induced seismicity and distinguishing between normal and anomalous earthquake diffusion are fundamental for predicting the essential characteristics of the system.

Pursuant to the latter, we presented the application of a purely stochastic framework based on the CTRW model and the TFDE to the spatiotemporal evolution of injection-induced seismicity in EGSs. The key quantity in the CTRW model is *ψ*(*x*,*t*), which in our case expresses the joint probability density for an earthquake occurring at position *x* after time *t*. Having defined *ψ*(*x*,*t*), the spatiotemporal evolution of seismicity can be determined without further assumptions with the appropriate master equation (see “Supplementary Methods”).

The applicability of the model was demonstrated for two case studies associated with hydraulic stimulations conducted in the Cooper Basin geothermal field in 2003 and 2012. In both cases, the MSD of induced seismicity away from the injection well scales as a fractional order power-law with time with diffusion exponents less than unity, signifying *subdiffusion* of induced seismicity. Subdiffusion is most commonly encountered in triggered earthquake diffusion despite the driving mechanism^20,36–43^, indicating that nonlinear inelastic relaxation of the crust occurs in response to a stress perturbation. However, the whole hierarchy of diffusion exponents reported manifests the variability of the physical process, and further studies are needed to decipher the exact details (triggering mechanism, geologic conditions, rheology, stress regime, etc.) that give rise to particular diffusion regimes.

Subdiffusion is further inferred from the power-law dependence of *ψ*(*x*,*t*) on time *t*. In both cases that we studied, the waiting-time distributions scale according to the *q*-generalized gamma function presenting asymptotic power-law behavior. The latter indicates long-term memory effects in the temporal evolution of injection-induced seismicity in the Habanero field, contrasting a random (Poissonian) occurrence. Furthermore, this scaling behavior implies that in the long-term limit, the characteristic waiting time *T* diverges, signifying the emergence of even longer waiting times between successive earthquakes. The latter suggests that sporadic earthquakes between long quiescent periods will continue to occur in the stimulated volume long after termination of injection, in agreement with observations from various EGS where induced earthquakes are observed years after hydraulic stimulation^18^.

Subdiffusion further implies that the concentration profile of seismicity is highly non-Gaussian but instead presents a skewed distribution, with the peak concentration close to the initial source of the stress perturbation (i.e., the injection well) slowly moving away with time. This is exactly the scaling behavior observed in both studied cases. For both Habanero-1 and Habanero-4, the peak earthquake occurrence is centered close to the two injection wells, while their spatial density gradually decays with increasing distance stretching out the concentration profiles. To model the observations, we used the TFDE, which is interchangeable with the subdiffusive regime of the CTRW model. In such cases of anomalous diffusion, fractional derivatives naturally arise to account for memory effects and long-range correlations often encountered in diffusion processes in complex heterogeneous media^44^. The asymptotic solution of the TFDE applied to the data shows good agreement, capturing the essential characteristics of earthquake occurrence. As might be expected, some discrepancy between the model and the data exists owing to the complexity of the process and to the nature of the modeling approach, as the theory is for an ensemble average, while the recorded seismicity represents only one “realization” responding to the hydraulic stimulation.

Goebel and Brodsky^19^ argued that the spatial occurrence of injection-induced seismicity is represented by two populations, the first showing an abrupt exponential decay associated with a dominant pore-pressure triggering mechanism and the second showing a power-law spatial decay associated with poroelastic effects. As previously discussed, the concentration profiles of seismicity in the Habanero field show a peak event concentration close to the two injection wells that spreads slowly with time and an exponential decay of seismicity away from the wells. The latter is in agreement with the results of^19^ regarding the spatial exponential decay of induced seismicity in the Habanero field and implies that the relaxation of fluid overpressures is the dominant triggering mechanism of induced seismicity in this case, in further agreement with Refs.^29,45^. In such cases, anomalous diffusion of injection-induced seismicity can emerge from a “non-Fickian” fluid transport process that channels along complex flow pathways with anisotropic hydraulic properties and within a heterogeneous and dynamically evolving permeability field that may vary over several orders of magnitude within the medium. Similar phenomena have been discussed for fluid flow in fractured media^46–48^ and fault damage zones^49^.

Finally, our results further suggest that the CTRW model can be directly incorporated into EGS-induced seismicity forecasts. Once the diffusion exponent of seismicity is defined, the model can make reasonable and computationally inexpensive predictions regarding the ensemble average of the spatial expansion of seismicity with time, an essential assessment for designing appropriate mitigation and regulatory strategies. This exact stochastic nature, which does not need to incorporate finer details of the system, has established the CTWR model during the last 5 decades quite compelling for modeling anomalous diffusion phenomena.

### Supplementary Information

Supplementary Information.

## Data Availability

Seismic event catalogues and hydraulic data for the hydraulic stimulations in the Habanero field (Cooper Basin, Australia) were kindly provided in a hard disk by the Department of State Development (SDS) of South Australia and are publicly available from EPOS Episodes Platform (https://episodesplatform.eu/?lang=en#episode:COOPER_BASIN).

## References

[1] Ellsworth, W. L. Injection-induced earthquakes. *Science* **341**, 1225942 (2013). 23846903 10.1126/science.1225942

[2] Weingarten, M., Ge, S., Godt, J. W., Bekins, B. A. & Rubinstein, J. L. High-rate injection is associated with the increase in US mid-continent seismicity. *Science* **348**, 1336–1340 (2015). 26089509 10.1126/science.aab1345

[3] Foulger, G. R., Wilson, M. P., Gluyas, J. G., Julian, B. R. & Davies, R. J. Global review of human-induced earthquakes. *Earth Sci. Rev.* **178**, 438–514 (2018).

[4] Raleigh, C. B., Healy, J. H. & Bredehoeft, J. D. An experiment in earthquake control at Rangely, Colorado. *Science* **191**, 1230–1237 (1976). 17737698 10.1126/science.191.4233.1230

[5] McGarr, A., Simpson, D. & Seeber, L. Case histories of induced and triggered seismicity. In *International Handbook of Earthquake & Engineering Seismology* Vol. 81A (eds Lee, W. *et al.*) 647–661 (Academic Press, 2002).

[6] McGarr, A. *et al.* Coping with earthquakes induced by fluid injection. *Science* **347**, 830–831 (2015). 25700505 10.1126/science.aaa0494

[7] Grigoli, F. *et al.* The November 2017 M_w_ 5.5 Pohang earthquake: A possible case of induced seismicity in South Korea. *Science* **360**, 1003–1006 (2018). 29700226 10.1126/science.aat2010

[8] Healy, J. H., Rubey, W. W., Griggs, D. T. & Raleigh, C. B. The Denver earthquakes. *Science* **161**, 1301–1310 (1968). 17831340 10.1126/science.161.3848.1301

[9] Pearson, C. The relationship between microseismicity and high pore pressures during hydraulic stimulation experiments in low permeability granitic rocks. *J. Geophys. Res.* **86**, 7855–7864 (1981).

[10] Segall, P. & Lu, S. Injection-induced seismicity: Poroelastic and earthquake nucleation effects. *J. Geophys. Res. Solid Earth* **120**, 5082–5103 (2015).

[11] Catalli, F., Rinaldi, A. P., Gischig, V., Nespoli, M. & Wiemer, S. The importance of earthquake interactions for injection-induced seismicity: Retrospective modeling of the Basel Enhanced Geothermal System. *Geophys. Res. Lett.* **43**, 4992–4999 (2016).

[12] Kwiatek, G. *et al.* Effects of long-term fluid injection on induced seismicity parameters and maximum magnitude in northwestern part of The Geysers geothermal field. *J. Geophys. Res. Solid Earth* **120**, 7085–7101 (2015).

[13] Guglielmi, Y., Cappa, F., Avouac, J. P., Henry, P. & Elsworth, D. Seismicity triggered by fluid injection–induced aseismic slip. *Science* **348**, 1224–1226 (2015). 26068845 10.1126/science.aab0476

[14] Bhattacharya, P. & Viesca, R. C. Fluid-induced aseismic fault slip outpaces pore-fluid migration. *Science* **364**, 464–468 (2019). 31048487 10.1126/science.aaw7354

[15] Keranen, K. M., Weingarten, M., Abers, G. A., Bekins, B. A. & Ge, S. Sharp increase in central Oklahoma seismicity since 2008 induced by massive wastewater injection. *Science* **345**, 448–451 (2014). 24993347 10.1126/science.1255802

[16] Goebel, T. H. W., Weingarten, M., Chen, X., Haffener, J. & Brodsky, E. E. The 2016 Mw5.1 Fairview, Oklahoma earthquakes: Evidence for long-range poroelastic triggering at >40 km from fluid disposal wells. *Earth Planet. Sci. Lett.* **472**, 50–61 (2017).

[17] Peterie, S. L., Miller, R. D., Intfen, J. W. & Gonzales, J. B. Earthquakes in Kansas induced by extremely far-field pressure diffusion. *Geophys. Res. Lett.* **45**, 1395–1401 (2018).

[18] Keranen, K. M. & Weingarten, M. Induced seismicity. *Annu. Rev. Earth Planet. Sci.* **46**, 149–174 (2018).

[19] Goebel, T. H. & Brodsky, E. E. The spatial footprint of injection wells in a global compilation of induced earthquake sequences. *Science* **361**, 899–904 (2018). 30166486 10.1126/science.aat5449

[20] Michas, G. & Vallianatos, F. Modelling earthquake diffusion as a continuous-time random walk with fractional kinetics: the case of the 2001 Agios Ioannis earthquake swarm (Corinth Rift). *Geophys. J. Int.* **215**, 333–345 (2018).

[21] Bouchaud, J. P. & Georges, A. Anomalous diffusion in disordered media: Statistical mechanisms, models and physical applications. *Phys. Rep.* **195**, 127–293 (1990).

[22] Shlesinger, M. F., Zaslavsky, G. M. & Klafter, J. Strange kinetics. *Nature* **363**, 31–37 (1993).

[23] Michas, G. & Vallianatos, F. Stochastic modeling of nonstationary earthquake time series with long-term clustering effects. *Phys. Rev. E* **98**, 042107 (2018).

[24] Tsallis, C. *Introduction to Nonextensive Statistical Mechanics: Approaching a Complex World* (Springer, 2009).

[25] Vallianatos, F., Papadakis, G. & Michas, G. Generalized statistical mechanics approaches to earthquakes and tectonics. *Proc. R. Soc. A* **472**, 20160497 (2016). 28119548 10.1098/rspa.2016.0497 PMC5247524

[26] Vallianatos, F. & Michas, G. Complexity of fracturing in terms of non-extensive statistical physics: From earthquake faults to Arctic sea ice fracturing. *Entropy* **22**, 1194 (2020). 33286962 10.3390/e22111194 PMC7712365

[27] Metzler, R. & Klafter, J. The random walk’s guide to anomalous diffusion: A fractional dynamics approach. *Phys. Rep.* **339**, 1–77 (2000).

[28] Helmstetter, A. & Sornette, D. Diffusion of epicenters of earthquake aftershocks, Omori’s law, and generalized continuous-time random walk models. *Phys. Rev. E* **66**, 061104 (2002). 10.1103/PhysRevE.66.061104 12513267

[29] Baisch, S., Weidler, R., Vörös, R., Wyborn, D. & de Graaf, L. Induced seismicity during the stimulation of a geothermal HFR reservoir in the Cooper Basin, Australia. *Bull. Seismol. Soc. Am.* **96**, 2242–2256 (2006).

[30] Baisch, S. *et al.* Continued geothermal reservoir stimulation experiments in the Cooper Basin (Australia). *Bull. Seismol. Soc. Am.* **105**, 198–209 (2015).

[31] Amorèse, D. Applying a change-point detection method on frequency-magnitude distributions. *Bull. Seismol. Soc. Am.* **97**, 1742–1749 (2007).

[32] Herrmann, M., Kraft, T., Tormann, T., Scarabello, L. & Wiemer, S. A consistent high-resolution catalog of induced seismicity in Basel based on matched filter detection and tailored post-processing. *J. Geophys. Res. Solid Earth* **124**, 8449–8477 (2019).

[33] Scher, H. & Montroll, E. W. Anomalous transit-time dispersion in amorphous solids. *Phys. Rev. B* **12**, 2455 (1975).

[34] Giardini, D. Geothermal quake risks must be faced. *Nature* **462**, 848–849 (2009). 20016577 10.1038/462848a

[35] Kwiatek, G. *et al.* Controlling fluid-induced seismicity during a 6.1-km-deep geothermal stimulation in Finland. *Sci. Adv.* **5**, eaav7224 (2019). 31049397 10.1126/sciadv.aav7224 PMC6494490

[36] Marsan, D., Bean, C. J., Steacy, S. & McCloskey, J. Spatio-temporal analysis of stress diffusion in a mining-induced seismicity system. *Geophys. Res. Lett.* **26**, 3697–3700 (1999).

[37] Marsan, D., Bean, C. J., Steacy, S. & McCloskey, J. Observation of diffusion processes in earthquake populations and implications for the predictability of seismicity systems. *J. Geophys. Res. Solid Earth* **105**, 28081–28094 (2000).

[38] Huc, M. & Main, I. G. Anomalous stress diffusion in earthquake triggering: correlation length, time dependence, and directionality. *J. Geophys. Res. Solid Earth* **108**, 2324 (2003).

[39] McKernon, C. & Main, I. G. Regional variations in the diffusion of triggered seismicity. *J. Geophys. Res. Solid Earth* **110**, 1–12 (2005).

[40] Helmstetter, A., Ouillon, G. & Sornette, D. Are aftershocks of large California earthquakes diffusing?. *J. Geophys. Res. Solid Earth* **108**, ESE9-1–24 (2003).

[41] Li, X., Main, I. & Jupe, A. Induced seismicity at the UK ‘hot dry rock’ test site for geothermal energy production. *Geophys. J. Int.* **214**, 331–344 (2018).

[42] Michas, G., Kapetanidis, V., Kaviris, G. & Vallianatos, F. Earthquake diffusion variations in the Western Gulf of Corinth (Greece). *Pure Appl. Geophys.* **178**, 2855–2870 (2021).

[43] Michas, G., Kapetanidis, V., Spingos, I., Kaviris, G. & Vallianatos, F. The 2020 Perachora peninsula earthquake sequence (East Corinth Rift, Greece): Spatiotemporal evolution and implications for the triggering mechanism. *Acta Geophys.* **70**, 2581–2601 (2022).

[44] Evangelista, L. R. & Lenzi, E. K. *Fractional Diffusion Equations and Anomalous Diffusion* (Cambridge University Press, 2018).

[45] Baisch, S. Inferring in situ hydraulic pressure from induced seismicity observations: An application to the Cooper Basin (Australia) geothermal reservoir. *J. Geophys. Res. Solid Earth* **125**, e2019JB019070 (2020).

[46] Berkowitz, B., Cortis, A., Dentz, M. & Scher, H. Modeling non-Fickian transport in geological formations as a continuous time random walk. *Rev. Geophys.* **44**, RG2003 (2006).

[47] Berkowitz, B. & Scher, H. Theory of anomalous chemical transport in random fracture networks. *Phys. Rev. E* **57**, 5858 (1998).

[48] O’Brien, G. S., Bean, C. J. & McDermott, F. A numerical study of passive transport through fault zones. *Earth Planet. Sci. Lett.* **214**, 633–643 (2003).

[49] Brixel, B. *et al.* Tracking fluid flow in shallow crustal fault zones: 2. Insights from cross-hole forced flow experiments in damage zones. *J. Geophys. Res. Solid Earth* **125**, e2019JB019108 (2020).

