# Supplementary material for: Stochastic modeling of injection induced seismicity based on the continuous time random walk model
Source: Sci Rep. 2024 Feb 28;14:4951. doi: 10.1038/s41598-024-55062-0 (PMC11316850; doi:10.1038/s41598-024-55062-0)
Supplement: Supplementary file 1 — Supplementary Information. [file 41598_2024_55062_MOESM1_ESM.pdf]

Supplementary Information

for

Stochastic modeling of injection induced seismicity based on the continuous time random walk model

Georgios Michas[1*] and Filippos Vallianatos[1,2]

[1] Section of Geophysics – Geothermics, Department of Geology and Geoenvironment, National and Kapodistrian University of Athens, Athens, Greece.

[2] Institute of Physics of Earth's Interior and Geohazards, UNESCO Chair on Solid Earth Physics and Geohazards Risk Reduction, Hellenic Mediterranean University Research Center, Crete, Greece.

* Correspondence to: gemichas@geol.uoa.gr

This PDF file includes:

1. Supplementary Methods

   1.1 The continuous time random walk (CTRW) model

   1.2 The time-fractional diffusion equation

2. Supplementary Figures S1 – S7

3. References

1. Supplementary Methods

1.1 The continuous time random walk (CTRW) model

The CTRW model was introduced by Ref. [1] and later by Ref. [2] as a generalization of the simple random walk model, in which a walker makes random steps on a discrete lattice at regular time intervals. In the continuous-time generalization of a random walk, fixed times are relaxed. Then, the spatial steps, or jumps, x and time steps, or waiting times, τ, between successive positions are considered as continuous random variables drawn from a joint probability density $\psi(x,t)$, which denotes the ensemble averaged probability of the walker making a transition from the previous site x_{n-1} to a new site x_n after waiting for some time τ_n between arrival times t ($\tau_n = t_n - t_{n-1}$). Analytic reviews of the CTRW theory can be found in Refs. [3,4]. In the following, we summarize the basic principles that apply in the CTRW model and are further used to form the modeling approach.

Within the CTRW context, we consider seismicity as a point process in time and space marked by the magnitude of the event. We also consider that, starting from the origin (x_0=0, t_0=0), seismicity undergoes a random walk in time and space, where each new event site is the new position of the random walk that occurs after waiting some time τ at the previous site. We describe this process with the joint probability density $\psi(x,t)$, which, in the case of seismicity, expresses the probability of an earthquake occurring at position x after time t. We also consider that the jump length and waiting time between successive earthquakes are independent random variables. This case corresponds to the decoupled form of $\psi(x,t)$, i.e., $\psi(x, t) = \varphi(t)\lambda(x)$, where $\lambda(x)$ and $\varphi(t)$ denote the probability densities of the jump lengths and waiting times, respectively.

Having defined $\psi(x,t)$, $\lambda(x)$ and $\varphi(t)$ can be deduced as:

$$\lambda(x) = \int_0^\infty \mathrm{d}t\psi(x,t) \tag{S1}$$

and

$$\varphi(t) = \int \mathrm{d}x\psi(x,t), \tag{S2}$$

where $\lambda(x)\mathrm{d}x$ is the probability of a jump length in the interval (x, x + dx) and $\varphi(t)\mathrm{d}t$ *is* the probability of a waiting time in the interval (t, t + dt). Along the lines of the CTRW theory, finite or infinite moments of $\varphi(t)$ and $\lambda(x)$ determine the type of the diffusive process. If the characteristic, or mean, waiting time $T = \int_0^\infty \mathrm{d}t\varphi(t)t$ and the jump length variance $\sigma^2 = \int_{-\infty}^{+\infty} \mathrm{d}x\lambda(x)x^2$ are finite, then the random walk in the long-time limit corresponds to the well-

known Brownian motion (normal diffusion). However, if T and σ^2 diverge, then anomalous diffusion emerges. In this case, $\lambda(x)$ and $\varphi(t)$ present heavy tails with asymptotic power-law scaling. For an infinite jump length variance σ^2, the so-called Lévy flights occur; however, for a finite σ^2 and infinite T, the random walk is characterized by long rest times, and the process corresponds to subdiffusion [3,4]. Also note that, apart from the finiteness or divergence of the moments T and σ^2, the details of $\lambda(x)$ and $\varphi(t)$ are irrelevant for the CTRW model and the diffusion process [5].

1.2 The time-fractional diffusion equation

Given the previous definitions of $\varphi(t)$, $\lambda(x)$ and $\psi(x,t)$, a CTRW process can be described by the generalized master equation [3,6,7]:

$$F(x,t) = \delta(x)\delta(t) + \int_{-\infty}^{+\infty} \mathrm{d}x' \int_0^{\infty} \mathrm{d}t' F(x',t')\psi(x-x',t-t'). \tag{S3}$$

The latter equation relates the pdf $F(x,t)$ with the pdf $F(x',t')$ of the random walker having just arrived at position x' at time t', weighted by the propagation function $\psi(x-x', t-t')$ that describes all possible steps from (x',t') to (x,t) [8]. The first summand of equation (S3) denotes the initial condition chosen to be at the origin ($x = 0$) at time $t = 0$, with δ being the Dirac delta function. The previous master equation is valid at any time and at all (one-, two- or three-dimensional) space scales.

Given the previous definition, the relationship between $F(x,t)$ and $P(x,t)$, i.e., the probability density function of the walker being at some position x after time t, also referred to as the propagator, is:

$$P(x,t) = \int_0^{t} \mathrm{d}t F(x,t')\Psi(t-t'), \tag{S4}$$

where

$$\Psi(t-t') = 1 - \int_0^{t-t'} \mathrm{d}t''\varphi(t'') \tag{S5}$$

is the cumulative probability for the walker not to jump in the time interval $[t', t]$. In terms of earthquake occurrence, $P(x,t)$ is the probability that an earthquake has occurred at x at time t' and that no other earthquake has occurred until time t ($t' \leq t$) [8].

To find the position of the propagator, the standard approach is the Laplace–Fourier transform [1]. If $\psi(k,u)$ is the Laplace–Fourier transform of $\psi(x,t)$ and $\lambda(k)$ and $\varphi(u)$ are the Fourier and Laplace transforms of $\lambda(x)$ and $\varphi(t)$, respectively, then the Laplace–Fourier transform of $F(x,t)$, i.e., $F(k,u)$, is given by:

$$F(k,u) = \frac{P_0(k,u)}{1-\psi(k,u)}, \tag{S6}$$

where $P_0(k,u)$ is the Laplace–Fourier transform of the initial condition $P_0(x,t) = \delta(x)\delta(t)$. From equations (S4) and (S6), the Laplace–Fourier transform $P(k,u)$ of $P(x,t)$ is given by:

$$P(k,u) = \frac{1-\varphi(u)}{u}\frac{P_0(k)}{1-\psi(k,u)}. \tag{S7}$$

From equation (S7), the various diffusion regimes can further be explored in the Laplace–Fourier domain. Let us recall that in the subdiffusive regime ($a < 1$), the jump length variance σ^2 is finite, while the characteristic waiting time T diverges. In this case, the Laplace transform of $\varphi(t)$ is given by:

$$\varphi(u) \sim 1 - (uT)^a, \tag{S8}$$

while the Fourier transform of $\lambda(x)$ is provided by:

$$\lambda(k) \sim 1 - \sigma^2 k^2. \tag{S9}$$

From equations (S7), (S8) and (S9), the Laplace–Fourier transform $P(k,u)$ for very small u and k becomes:

$$P(k,u) = \frac{[P_0(k,u)/u]}{1+K_a u^{-a} k^2}. \tag{S10}$$

From equation (S10) and by applying the integration rule for fractional integrals, the time-fractional diffusion equation (TFDA) can be derived (see also [4]):

$$\frac{\partial}{\partial t}P(x,t) = {}_0D_t^{1-a} K_a \frac{\partial^2}{\partial x^2}P(x,t), \tag{S11}$$

where ${}_0D_t^{1-a}$ is the Riemann–Liouville fractional operator of order $1-a$ ($0 < a < 1$) applied to $P(x,t)$ in terms of convolution (e.g., [9,10]):

$${}_0D_t^{1-a}P(x,t) = \frac{1}{\Gamma(a)}\frac{\partial}{\partial t}\int_0^t dt' \frac{P(x,t')}{(t-t')^{1-a}}. \tag{S12}$$

In equations (S10) and (S11), K_a is the generalized diffusion coefficient defined as $K_a \equiv \sigma^2/T^a$, taking the dimensions of σ and T, i.e., $[K_a] = m^2/s^\alpha$ [4].

In the limit $a \rightarrow 1$, the standard diffusion equation is recovered from the TFDA:

$$\frac{\partial}{\partial}P(x,t) = K_1 \frac{\partial^2}{\partial x^2}P(x,t), \tag{S13}$$

where K_1 is the diffusion coefficient expressed in integer dimensions m^2/s. Hence, K_a can be considered the fractional counterpart of K_1, and in the subdiffusive regime, where $0 < a < 1$, K_a

is always less than K_1. As a result of the central limit theorem (CLT), in the long-term limit and for the initial condition of the walker being at the origin (x=0) at time t=0, the solution of the diffusion equation (equation (S13)) is the standard Gaussian distribution:

$$P(x,t) = \frac{1}{\sqrt{4\pi K_1 t}} \exp\left(-\frac{x^2}{4K_1 t}\right). \qquad \text{(S14)}$$

From equation (S14), one can immediately verify that the variance of the Gaussian propagator, or in other words, the MSD $\langle x^2(t)\rangle$, is linear with t (e.g., [5]). Additionally, note that the diffusion equation (equation (S13)) is equivalent to Fick's second law; therefore, in many cases, this regime is referred to as Fickian diffusion, particularly when particle or fluid transport in the subsurface is involved (e.g., [11]).

The solution of the TFDA (equation (S11)) can be approached by using the Fox hypergeometric functions, providing the asymptotic behavior of the propagator $P(x,t)$ for large x [4,12]. A similar approach can be used to find the position of the propagator in terms of the pdf $F(x,t)$. In particular, Ref. [8] showed that $F(x,t)$ is related to $P(x,t)$ as:

$$F(x,t) = (\tau')^{-a}{}_0D_t^{1-a}P(x,t), \qquad \text{(S15)}$$

where ${}_0D_t^{1-a}$ is the Riemann–Liouville operator and $\tau' = T\big(\Gamma(1-a)\big)^{1/a}$ ($a < 1$). Using the Riemann–Liouville derivation, the asymptotic behavior of $F(x,t)$ can be written in terms of Fox hypergeometric functions as [8]:

$$F(x,t) \sim \frac{(\tau\prime)^{-a}}{2\sqrt{K_a}t^{1-(a/2)}} H_{1,1}^{1,0}\left[\frac{|x|}{\sqrt{K_a}t^{a/2}} \begin{matrix}(a/2,a/2)\\(0,1)\end{matrix}\right]. \qquad \text{(S16)}$$

From equation (S16) and by employing once again the standard theorem of Fox hypergeometric functions, the asymptotic behavior of $F(x,t)$ for large x $\left(x > \sqrt{K_a t^a}\right)$ can be derived [8]:

$$F(x,t) \sim \frac{(\tau')^{-a}}{\sqrt{K_\alpha}t^{1-(a/2)}} \left(\frac{|x|}{\sqrt{K_a t^a}}\right)^{d(1-a)/(2-a)} \times \exp\left[-\left(1-\frac{a}{2}\right)\left(\frac{a}{2}\right)^{a/(2-a)}\left(\frac{|x|}{\sqrt{K_a t^a}}\right)^{2/(2-a)}\right], \qquad \text{(S17)}$$

where d are the spatial dimensions.

2. Supplementary Figures S1 – S7

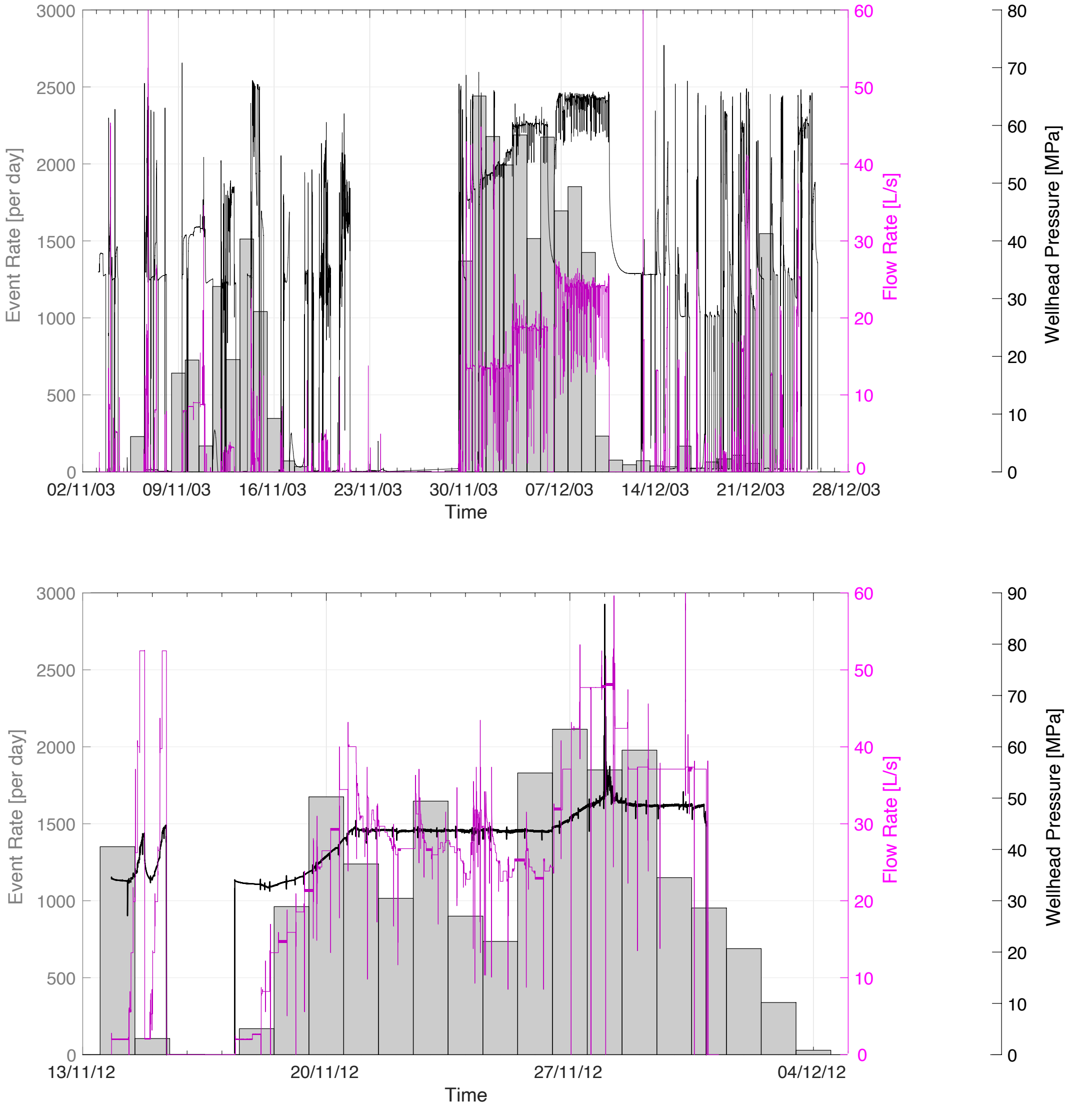

Figure S1. The daily rate of induced events, the injection rate and the wellhead pressure with time during hydraulic stimulation of the Habanero-1 (top) and Habanero-4 (bottom) wells.

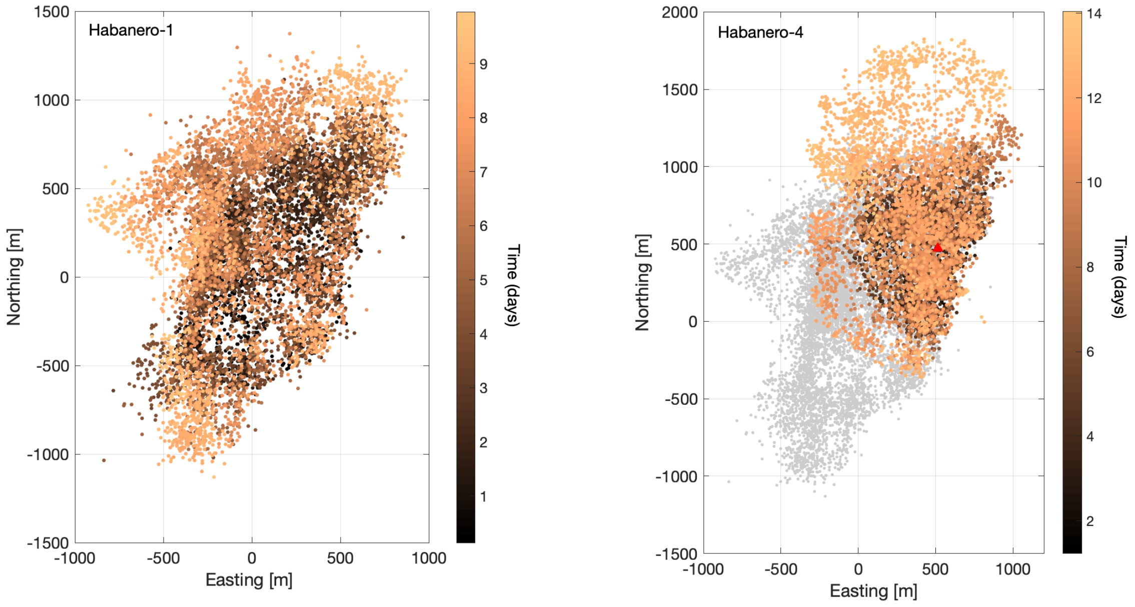

Figure S2. Spatial distribution of induced seismicity during the main hydraulic stimulation of the Habanero-1 (left) and Habanero-4 (right) wells, centered to the Habanero-1 well. The grey dots in the right panel show the distribution of seismicity during the main stimulation of the Habanero-1 well, while the red triangle shows the location of the Habanero-4 well. The color bar indicates the occurrence time of seismicity, counting in days since initiation of injection.

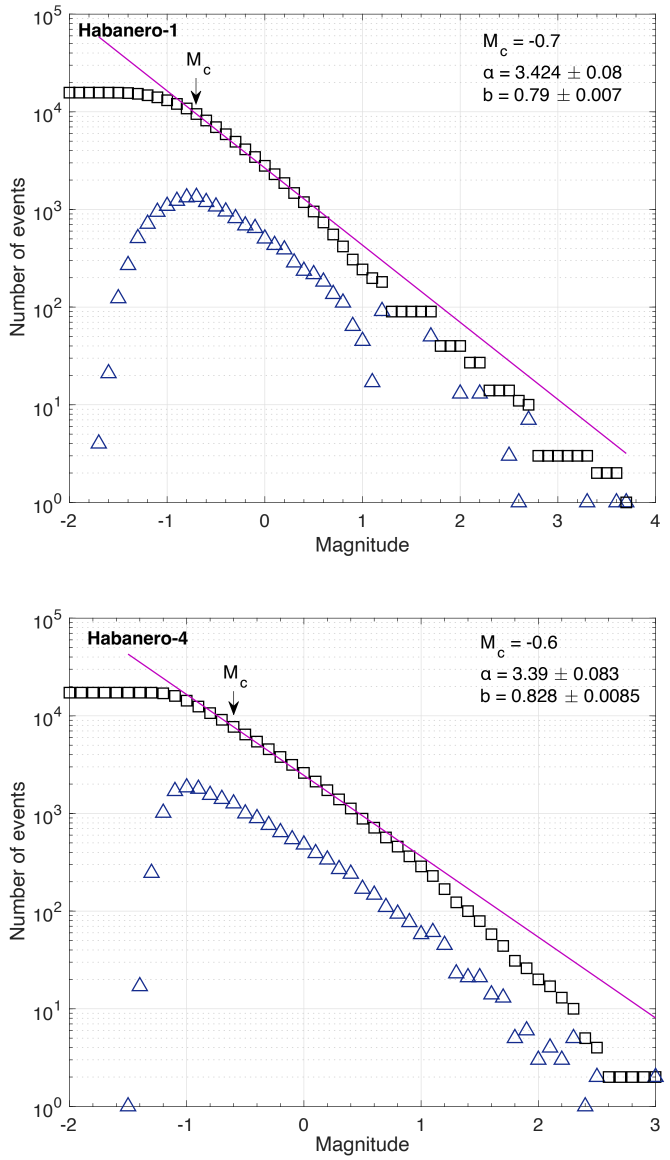

Figure S3. Frequency–magnitude distributions of seismicity that was induced during the main stimulation of the Habanero-1 (top) and Habanero-4 (bottom) wells, represented by the cumulative (squares) and the discrete (triangles) number of events. The magnitude of completeness (M_c) was estimated using the median-based analysis of the segment slope. The solid lines represent the Gutenberg–Richter relation for the *a* and *b* values shown in the legend.

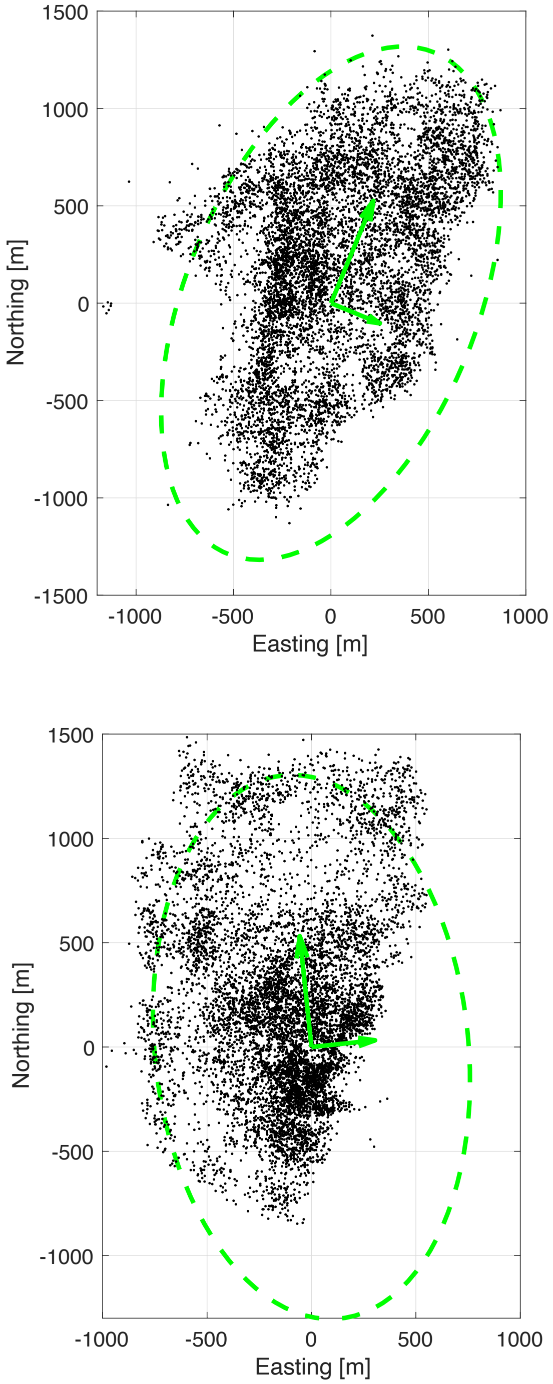

Figure S4. Spatial distribution of induced seismicity in 2D (black dots) during the main stimulation of the Habanero-1 (top) and Habanero-4 (bottom) wells, centered to the two wells. The arrows represent the principal components of the seismicity clouds derived with the principal component analysis, pointing at the direction of maximum spatial variance. The principal components define the principal axes of an ellipse (dashed green line) that includes 95% of all induced events.

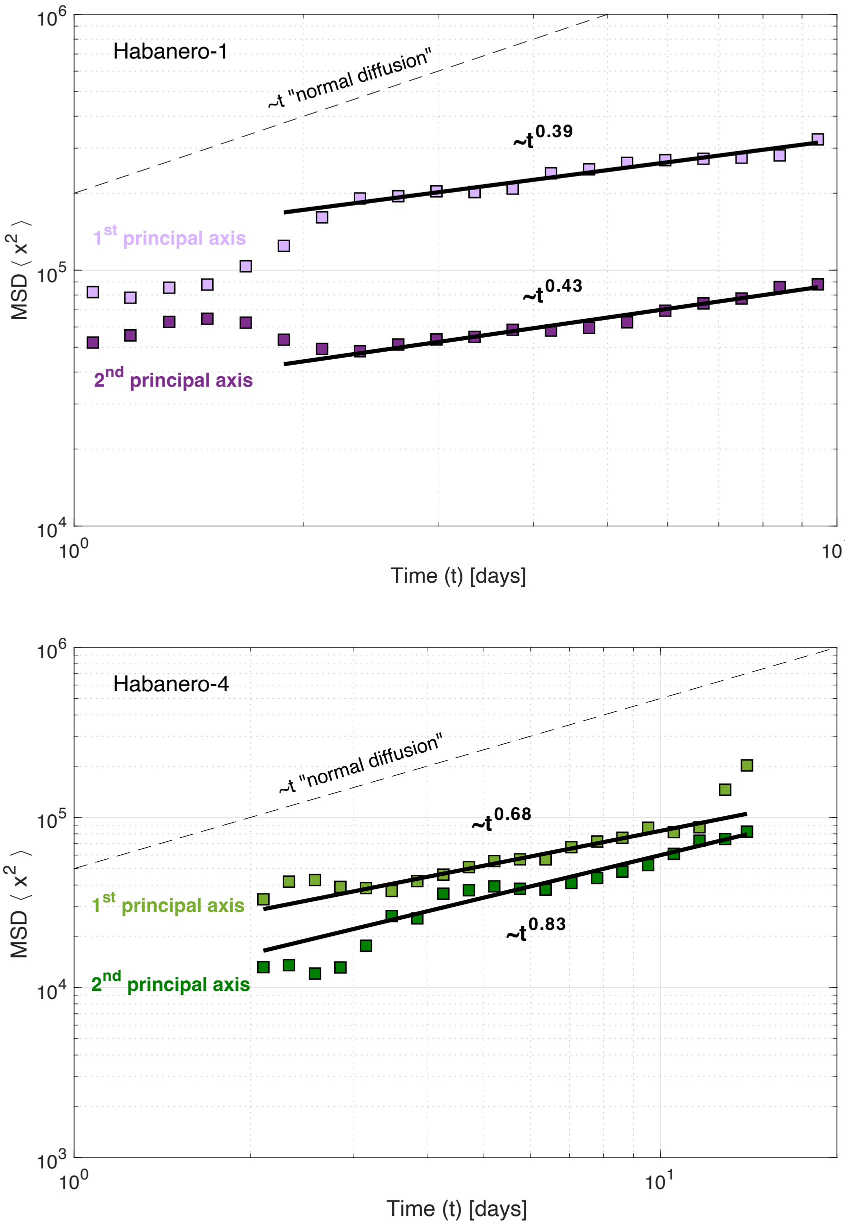

Figure S5. MSD of induced seismicity with time (filled squares) for Habanero-1 (top) and Habanero-4 (bottom) in logarithmically spaced bins and on double logarithmic axes. MSD is calculated by taking the 1D distances of the events from the origin along the principal axes of the ellipse that best describes the seismicity cloud (see Fig. S4). Solid lines represent the best-fiiting solutions according to the power law relationsip of equation (2). The dashed line represents the trend (a = 1) for normal diffusion.

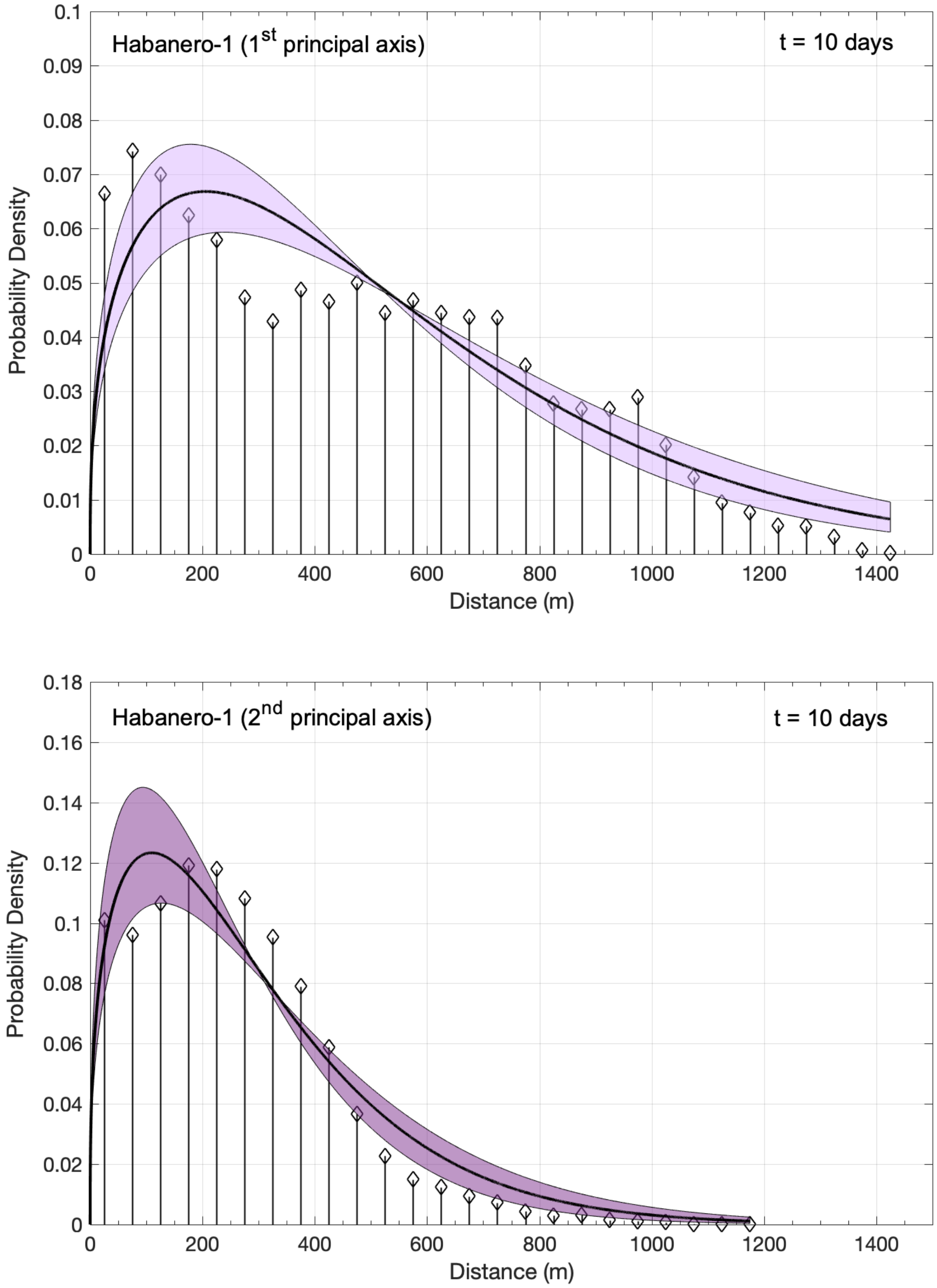

Figure S6. Normalized probability density of the absolute 1D distances between the induced events and the origin along the first (top) and second (bottom) principal axes of an ellipse that best fits the seismicity cloud during the main stimulation of the Habanero-1 well, represented as stem plots for t=10 days. The solid lines and the shaded areas indicate the asymptotic solution of the TFDE (equation (S17)) and the corresponding confidence intervals, respectively, for $K_a = 43.40 \cdot 10^3 \pm 10.57 \cdot 10^3$ (top) and $K_a = 11.32 \cdot 10^3 \pm 3.04 \cdot 10^3$ (bottom).

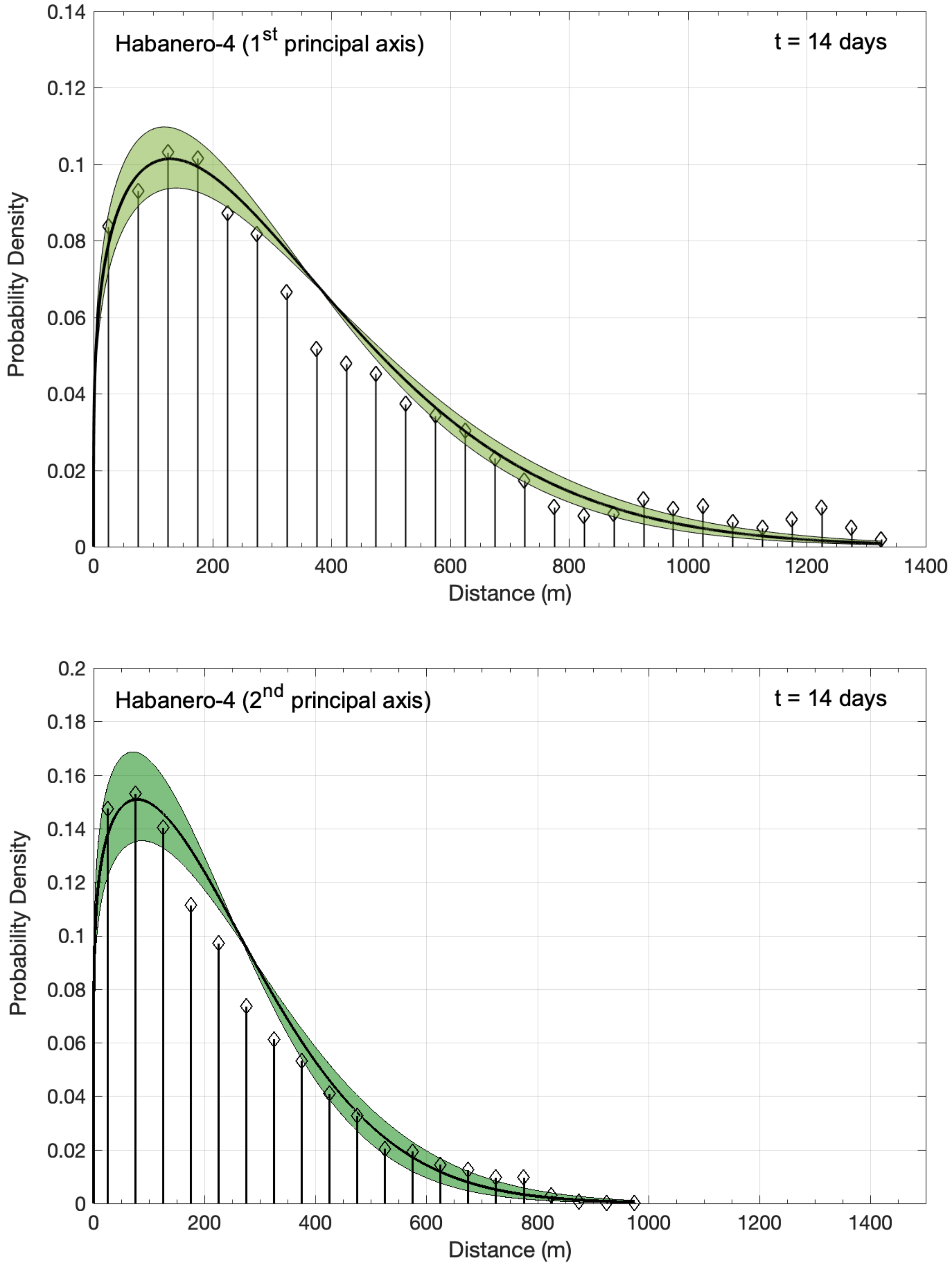

Figure S7. Normalized probability density of the absolute 1D distances between the induced events and the origin along the first (top) and second (bottom) principal axes of an ellipse that best fits the seismicity cloud during the main stimulation of the Habanero-4 well, represented as stem plots for t=14 days. The solid lines and the shaded areas indicate the asymptotic solution of the TFDE (equation (S17)) and the corresponding confidence intervals, respectively, for $K_a = 8.57 \cdot 10^3 \pm 1.23 \cdot 10^3$ (top) and $K_a = 3.12 \cdot 10^3 \pm 0.60 \cdot 10^3$ (bottom).

3. References

[1] Montroll, E. W. & Weiss, G. H. Random walks on lattices. II. *J. Math. Phys.* **6,** 167–181 (1965).

[2] Scher, H. & Montroll, E. W. Anomalous transit-time dispersion in amorphous solids. *Phys. Rev. B* **12,** 2455 (1975).

[3] Bouchaud, J. P. & Georges, A. Anomalous diffusion in disordered media: statistical mechanisms, models and physical applications. *Phys. Rep.* **195,** 127–293 (1990).

[4] Metzler, R. & Klafter, J. The random walk's guide to anomalous diffusion: a fractional dynamics approach. *Phys. Rep.* **339,** 1–77 (2000).

[5] Metzler, R., Jeon, J. H., Cherstvy, A. G., & Barkai, E. Anomalous diffusion models and their properties: non-stationarity, non-ergodicity, and ageing at the centenary of single particle tracking. *Phys. Chem. Chem. Phys.* **16,** 24128–24164 (2014).

[6] Klafter, J. & Silbey, R. Derivation of the continuous-time random-walk equation. *Phys. Rev. Lett.* **44,** 55 (1980).

[7] Hughes, B. D. *Random Walks and Random Environments* (Oxford University Press, 1995).

[8] Helmstetter, A. & Sornette, D. Diffusion of epicenters of earthquake aftershocks, Omori's law, and generalized continuous-time random walk models. *Phys. Rev. E* **66,** 061104 (2002).

[9] Miller, K. S. & Ross, B. *An Introduction to the Fractional Calculus and Fractional Differential Equations* (Wiley, 1993).

[10] Sokolov, I. M., Klafter, J. & Blumen, A. Fractional kinetics. *Physics Today* **55,** 48–54 (2002).

[11] Berkowitz, B., Cortis, A., Dentz, M. & Scher, H. Modeling non-Fickian transport in geological formations as a continuous time random walk. *Rev. Geophys*. **44,** RG2003 (2006).

[12] Barkai, E., Metzler, R. & Klafter, J. From continuous-time random walks to the fractional Fokker-Planck equation. *Phys. Rev. E* **61,** 132–138 (2000).
